# Time to return of spontaneous circulation and associations with early neurological outcome at hospital discharge after out-of-hospital cardiac arrest

**DOI:** 10.1007/s00392-026-02940-4

**Published:** 2026-06-03

**Authors:** Ulrik Rose, Marie Wulf, Morten Steen Kvistholm Jensen, Emma Riis Meyer, John Bro-Jeppesen

**Affiliations:** 1 Department of Cardiology, Aarhus University Hospital, Palle Juul-Jensens Boulevard 99, 8200 Aarhus N Aarhus, Denmark; 2 Department of Clinical Medicine, Aarhus University, Aarhus, Denmark

**Keywords:** Out-of-hospital cardiac arrest, Neurological function, Outcome, Cardiopulmonary resuscitation, MRS, MoCA, ROSC, Neurological recovery

## Abstract

**Aim:**

To assess the impact of time to return of spontaneous circulation (ROSC) on early in-hospital neurological function and long-term survival in survivors after out-of-hospital cardiac arrest (OHCA).

**Methods:**

This retrospective cohort study included survivors of OHCA admitted to the cardiology ward at Aarhus University Hospital. Patients were included from June 2020 to February 2024 and data were collected through review of medical records. Primary outcome was neurological function, and patients were stratified by modified Rankin Scale (mRS) at hospital discharge: good neurological outcome (mRS 0–2) and poor neurological outcome (mRS 3–6). Secondary outcomes were cognitive function assessed with Montreal Cognitive Assessment (MoCA) at hospital discharge and 180-day survival.

**Results:**

In total, 321 patients were included, of which 232 (72%) had good neurological outcome and 89 (28%) had poor neurological outcome. Every 5-min increase in time to ROSC was associated with decreased odds of good neurological outcome (OR 0.88, 95% CI 0.81–0.95, *p* < 0.0001). This association remained significant after adjusting for age and primary rhythm (OR_adjusted_ 0.86, 95% CI 0.78–0.95, *p* = 0.004). Patients with good neurological outcome had significantly higher MoCA scores (median 23 vs. 18, *p* < 0.001). In-hospital mortality was 6% and time to ROSC was not significantly associated with 180-day mortality.

**Conclusion:**

In initial OHCA survivors transferred to the cardiology ward, increased time to ROSC was independently associated with decreased odds of good neurological function at hospital discharge, even after adjusting for age and primary rhythm. The majority of patients had good neurological function at hospital discharge. In this population time to ROSC was not associated with mortality.

**Graphical Abstract:**

Figure created by the authors using Microsoft PowerPoint.

**Figure:**
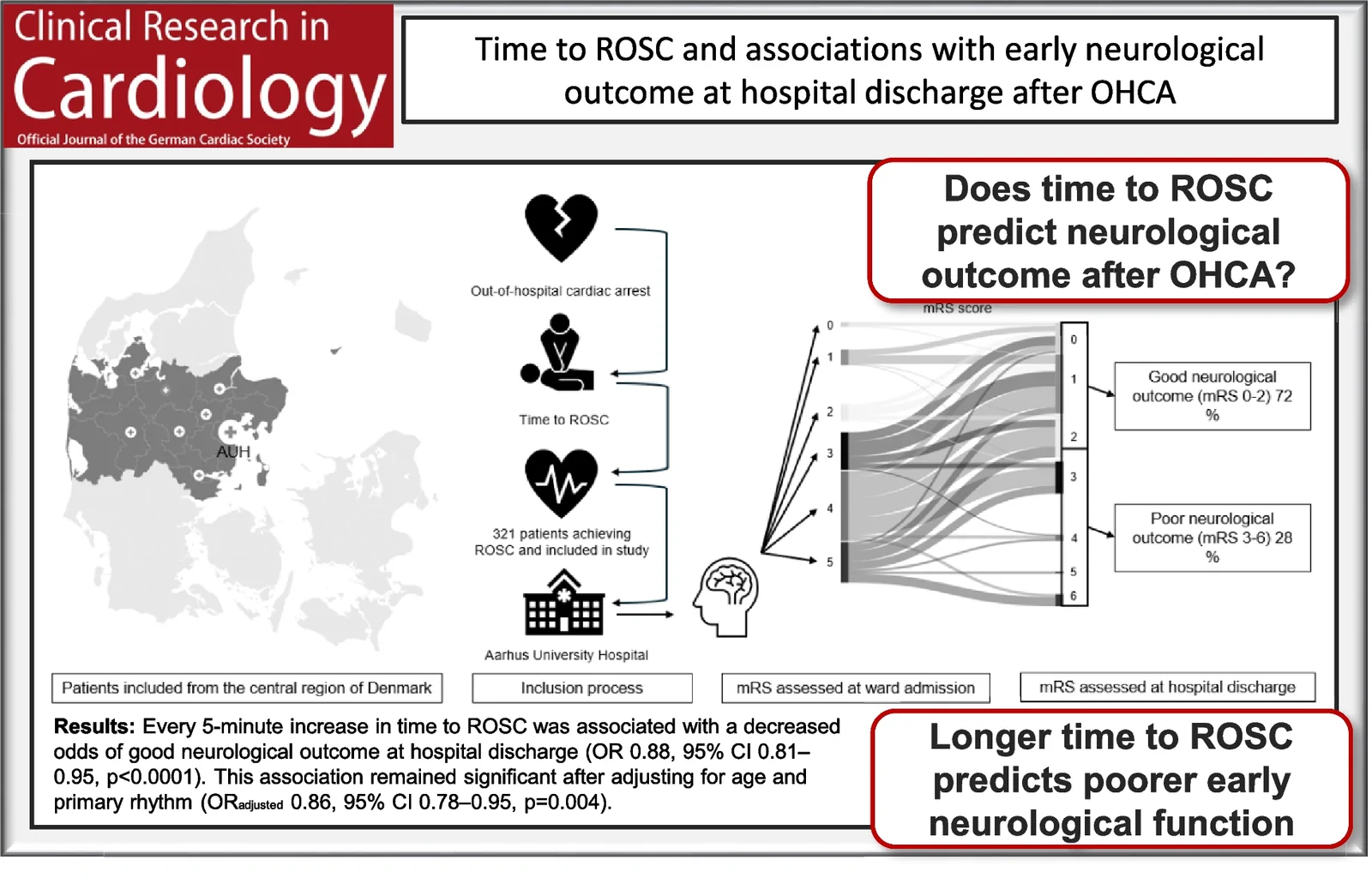

## Introduction

In 2023, the incidence of out-of-hospital cardiac arrest (OHCA) in Denmark was approximately 4,500 cases, corresponding to 78 cases of OHCA per 100,000 inhabitants [1]. Improved management of OHCA and growing public awareness has contributed to a four-fold increase in 30-day survival rate reaching 14.4%, compared to two decades before [1]. As survival rates improve, there is an emergent need for a better understanding of the neurological outcome among OHCA survivors. Neurological impairment is among the most common complications following OHCA [2] and one of the most critical factors influencing neurological outcome is the duration of global cerebral ischemia, closely related to time to return of spontaneous circulation (ROSC) [3, 4]. Despite its central role in pathophysiology, the association between time to ROSC and early in-hospital neurological function remains inadequately explored. Several OHCA studies have reported neurological status on survivors [5] but only a limited number have specifically examined its association with the duration of time to ROSC and neurological function during hospital admission and at hospital discharge. A systematic review [6] suggests that shorter time to ROSC is associated with better neurological outcomes at discharge, but findings are inconsistent and largely based on small or single-center cohorts. Consequently, more evidence is needed to clarify how the duration of time to ROSC impacts early neurological recovery.

This study aims to investigate the association between time to ROSC and temporal changes in early neurological function assessed at cardiology ward admission and hospital discharge in survivors after OHCA.

## Materials and methods

### Study area and population

This study cohort consists of adult patients, aged ≥ 18 years, admitted to Department of Cardiology, Aarhus University Hospital (AUH) after successful or ongoing resuscitation for OHCA with a presumed cardiac cause in the period between May 2020 and February 2024. In this study, patients were defined as initial OHCA survivors, if surviving to admission to the cardiology ward. Patients were included at the time of transfer from the ICU or coronary care unit to the cardiology ward. The cardiology ward functions as a monitored step-down unit where patients are hemodynamically stable and no longer require invasive ventilation or continuous vasoactive support. Patients who had in-hospital cardiac arrest, died during transportation, in the ICU or during initial treatment in the coronary care unit were excluded. Department of Cardiology, AUH is a tertiary referral center for cardiology and receives OHCA patients with unknown or presumed cardiac cause of cardiac arrest from the central region of Denmark with a population of 1.373.799 people [7]. Facilities such as transvenous pacing, coronary interventions, extracorporeal membrane oxygenation, right and left ventricular assist devices and targeted temperature management (TTM) were available 24/7 as part of a high-volume cardiac arrest center. Post-cardiac arrest care followed contemporary guidelines in regard to intensive care monitoring including TTM where the strategy changed on august 1 st 2022 to maintain normothermia. Acute coronary angiography was performed in all patients with ST-elevation in primary electrocardiogram or hemodynamic unstable, whereas a delayed coronary strategy was applied in other patients. Patients were typically transferred directly to the cardiology ward after stabilization or after completion of the post-resuscitation care in the ICU.

### Data collection

This study is a retrospective cohort study with patients transferred to the cardiology ward from coronary unit or intensive care unit (ICU) at AUH after initial care of OHCA. Patient data were prospectively included in an observational registry as part of a clinical quality project. The registry was approved by department and hospital administrations with a waiver of informed consent according to Danish legislation and aggregated data can be reported without further approvals by hospital staff. The retrospective data were obtained by review of medical records. The medical records consist of records from the emergency team in the pre-hospital setting, along with the medical records made by physicians at the hospital. The following variables were collected or assessed through medical records: time to ROSC, bystander CPR, witnessed arrest, primary rhythm, length of hospital stay, modified Rankin Scale (mRS) score, Montreal Cognitive Assessment (MoCA) score and survival.

OHCA, ROSC, and low-flow and no-flow time were defined according to the 2024 Utstein guidelines [8]. Time to ROSC was derived from prehospital emergency medical service (EMS) documentation supplemented by review of hospital medical records. In cases where ROSC occurred before rhythm analysis, time of ROSC was recorded according to EMS documentation. Implausible or missing time intervals were excluded and no multiple imputation was performed. Unwitnessed cardiac arrest was defined if the patient was not observed as conscious for more than 2 min before collapse. In patients experiencing recurrent cardiac arrest after initial ROSC, time to ROSC was defined as the cumulative duration of resuscitation efforts until sustained ROSC was achieved.

A volunteer responder is a CPR-trained civilian dispatched using global positioning system (GPS) coordinates to link the responder’s location with that of the cardiac arrest [9].

The primary heart rhythm was categorized as a shockable rhythm if it was ventricular fibrillation, ventricular tachycardia, or shocked by an AED. If it was asystole or pulseless electrical activity, it was classified as a non-shockable rhythm and “other” if it was a clinical cardiac arrest and the patient obtained ROSC before a heart rhythm was analyzed.

### Outcome

#### Modified Rankin Scale

The neurological function was assessed by mRS at the time of transfer from ICU to cardiology ward and at hospital discharge. The mRS is a disability ranking system which is divided into 6 subclasses of overall function with scores from 0 to 6, with 6 being death [10]. Good neurological outcome was defined as mRS 0–2 and poor neurological outcome was defined as mRS 3–6. mRS was assessed at the time of transfer from ICU to cardiology ward by nurses at the time of transfer, whereas mRS at hospital discharge was based on medical records. At hospital discharge, nursing staff routinely complete a structured discharge status describing the patient’s functional level and degree of independence, including the amount of assistance required in daily activities and whether support services are needed after discharge. To determine the most appropriate mRS category at discharge, we used the nursing discharge status together with the physician’s discharge summary. The mRS score was assessed by the authors (MW and UR) and was evaluated for inter-observer variation by a senior consultant (JBJ).

#### MoCA

Cognitive function was assessed using the MoCA, a 30-point screening assessment for detecting cognitive impairment recommended for use in survivors after OHCA. A score ≥ 26 is considered as a normal score [11]. The MoCA test was performed by a trained occupational therapist at the cardiology ward as close to hospital discharge as possible. MoCA testing was performed when patients were able to cooperate with the assessment. In cases where testing could not be performed, the data were recorded as missing.

#### Survival

Survival was defined as being alive at the time of hospital discharge and was determined from electronic hospital records. Long-term survival was assessed at 180 days post-arrest using data from the Danish Civil Registration System.

### Statistics

Data are presented as mean ± standard deviation (SD) or proportions (%), and differences were assessed by Wilcoxon rank-sum test or χ2-test, as appropriate. Variables with a non-normal distribution are presented as median with lower and upper quartiles (Q1–Q3) and differences were assessed by the Wilcoxon rank-sum test. Associations between time to ROSC and neurological outcome were analysed by logistic regression analysis and data presented as odds ratio (OR) with 95% confidence intervals (CI) and *p*-values. To quantify the temporal relationship, ORs were calculated per 5-min increase in time to ROSC. Time to ROSC was chosen as a composite marker of resuscitation time interval for use in the multivariable model as collinearity was identified between no-flow, low-flow and time to ROSC. To create a multivariable logistic regression analysis, the following variables were entered in the multivariable model: age, sex, witnessed arrest, bystander CPR, primary rhythm, and time to ROSC. Following the backward elimination procedure, the final model consisted of time to ROSC, age at arrest and shockable rhythm. Quartiles of time to ROSC defined four strata for survival analysis by Kaplan–Meier plots, and differences were tested by the log-rank test. One-hundred eighty-day mortality was investigated by unadjusted and adjusted Cox proportional-hazards models after checking for underlying assumptions of linearity, proportionality and lack of interactions. To create a multivariable Cox regression analysis, the following variables were entered in the multivariable model: age, sex, witnessed arrest, bystander CPR, primary rhythm, and time to ROSC. Following the backward elimination procedure, the final model consisted of bystander CPR and age at arrest. Results are presented as hazard ratios (HR) with 95% confidence intervals (CI) and *p*-values.

Inter-observer analysis of mRS scores was assessed by weighted Cohen’s kappa. A *p*-value < 0.05 was considered statistically significant. All statistical analyses were performed using the SAS statistical software, version 9.4 (SAS Institute 9.4, Cary, NC, USA).

## Results

A total of 328 patients were included in the registry. Of these patients, 7 were excluded from the analysis as they did not meet the inclusion criteria.

The majority of the 321 patients included in the study were discharged from the cardiology department with a good neurological outcome (72.3% vs. 27.7%). In the group with a good neurological outcome (*n* = 232), 54 (23%) had a mRS score of 0, 104 (45%) with mRS score of 1, and 74 (32%) with mRS score of 2.

In the group with a poor neurological outcome (*n* = 89), 56 (63%) had a mRS score of 3, 11 (12%) with mRS score of 4, only 3 (3%) had a mRS score of 5, and 19 (21%) died prior to discharge and thus had a mRS score of 6. The results of the inter-observer analysis on the assigned mRS scores showed a weighted Cohen’s kappa score of 0.73 (95% CI 0.54–0.92).

Baseline characteristics stratifying the study population into two groups based on a good neurological outcome (mRS 0–2) and poor neurological outcome (mRS 3–6) are shown in Table 1. The median time to ROSC was significantly increased in the poor neurological outcome group compared to the good neurological outcome group (15 vs. 13 min, *p* = 0.009) and similar results were found in terms of increased no-flow time (1 vs. 0 min, *p* = 0.004), respectively.

**Table 1:** Baseline characteristics

	All	Good neurological outcome	Poor neurological outcome	***p***-value
	*n* = 321	*n* = 232	*n* = 89	
Age, years (mean ± SD)	64 ± 14	62 ± 14	71 ± 11	< 0.0001
Male sex (%)	267 (83)	243 (83)	74 (83)	0.99
Primary rhythm				
Shockable (%)	279 (87)	210 (91)	69 (79)	0.009
Non-shockable (%)	28 (9)	12 (5)	16 (18)	
Other (%)	12 (4)	10 (4)	2 (2)	
Witnessed arrest (%)	288 (90)	211 (91)	77 (87)	0.25
Bystander CPR (%)	258 (89)	193 (91)	65 (82)	0.02
Time to ROSC, min, (Q1–Q3)	14 (9–20)	13 (8–20)	15 (10–29)	0.009
No-flow time, min (Q1–Q3)	0 (0–2)	0 (0–2)	1 (0–4)	0.004
Low-flow time, min (Q1–Q3)	12 (7–20)	12 (7–19)	14 (8–27)	0.08
AED applied (%)	118 (37)	89 (38)	29 (33)	0.19
LUCAS applied (%)	63 (20)	39 (17)	24 (27)	0.10
Defibrillation by prehospital team (%)	241 (76)	177 (77)	64 (74)	0.53
Length of hospital stay, days (Q1–Q3)	14 (10–20)	14 (10–18)	16 (11–26)	0.03
Volunteer responder assisted (%)	100 (31)	76 (33)	24 (27)	0.44

Data are presented as mean ±SD or median and lower and upper quartiles (Q1–Q3) as appropriate. The *p*-value is unadjusted and represents comparison between groups; good neurological outcome (mRS 0–2) and poor neurological outcome (mRS 3–6)

*mRS* modified Rankin Scale, *SD* standard deviation, *CPR* cardiopulmonary resuscitation, *ROSC* return of spontaneous circulation, *Q1–Q3* lower and upper quartile, *AED* automated external defibrillator, *LUCAS* Lund University Cardiopulmonary Assist System

### Primary outcome

Table 2 provides a full overview of the results of the univariate and multivariable logistic regression.

**Table 2:** Univariable and multivariable logistic regression analysis of a good neurological outcome

	**Univariable**		**Multivariable**	
	OR (95% CI)	*p*-value	OR (95% CI)	*p*-value
Age at arrest every 5 years increase	0.75 (0.66–0.84)	< 0.0001	0.70 (0.59–0.80)	< 0.0001
Gender female vs. male	1.00 (0.53–1.96)	0.99		
Primary rhythm				
Shockable vs. non-shockable	4.06 (1.84–9.18)	0.001	3.56 (1.27–10.52)	0.025
Witnessed arrest, yes vs. no	1.73 (0.78–3.69)	0.98		
Bystander CPR	2.60 (1.19–5.63)	0.98		
Volunteer responder, yes vs. no	1.31 (0.76–2.29)	0.44		
AED applied, yes vs. no	1.24 (0.74–2.11)	0.23		
Time to ROSC every 5 min increase	0.88 (0.81–0.95)	< 0.0001	0.86 (0.78–0.95)	0.004
Low-flow time, every 1 min increase	0.98 (0.96–0.99)	< 0.0001		
No-flow time, every 1 min increase	0.88 (0.80–0.97)	< 0.0001		

Good neurological outcome was defined as mRS 0–2 and poor neurological outcome was defined as mRS 3–6. Data are presented as odds ratios (OR) along with 95% confidence intervals (CI) and *p*-values

The following variables were entered in the multivariable model: age, sex, witnessed arrest, bystander CPR, primary rhythm, and time to ROSC. Following the backward elimination procedure, the final model consisted of time to ROSC, age at arrest and shockable rhythm

*mRS* modified Rankin Scale, *CPR* cardiopulmonary resuscitation, *ROSC* return of spontaneous circulation, *AED* automated external defibrillator

The univariable logistic regression analysis showed that for every 5-min interval the odds of a good neurological outcome at hospital discharge decreased by 12% (OR 0.88, 95% CI 0.81–0.95, *p* < 0.0001). Additionally, when assessing the low-flow and no-flow times, the odds decreased by 2% for every minute of low-flow time (OR 0.98, 95% CI 0.96–0.99, *p* < 0.0001) and by 12% for every minute of no-flow time (OR 0.88, 95% CI 0.80–0.97, *p* < 0.0001). The odds for a good neurological outcome were significantly associated with age and decreased by 25% (per 5 years, OR 0.75, 95% CI 0.66–0.84, *p* < 0.0001). A shockable rhythm was associated with a better neurological outcome with an OR of 4.06 (95% CI 1.84–9.18, *p* = 0.001).

We found no significant association between gender, witnessed cardiac arrest, bystander CPR, volunteer responder and use of AED in terms of early neurological outcome.

The results were consistent when adjusting for confounding factors and showed that for every 5-min increase in time to ROSC the odds of a good neurological outcome decreased by 14% (OR_adjusted_ 0.86 (95% CI 0.78–0.95), *p* = 0.004). A shockable rhythm increased the odds of a good neurological outcome (OR 3.56 (95% CI 1.27–10.52, *p* = 0.025)), while higher age at arrest decreased the odds (OR 0.70 per 5-year increase (95% CI 0.59–0.80, *p* < 0.001)), Table 2.

The median time from cardiac arrest to admission to the cardiology ward was 2 days (Q1–Q3, 2–4). Longer ICU stay was associated with lower odds of good neurological outcome (OR 0.87 per day, 95% CI 0.81–0.92, *p* < 0.0001).

**Fig. 1:**
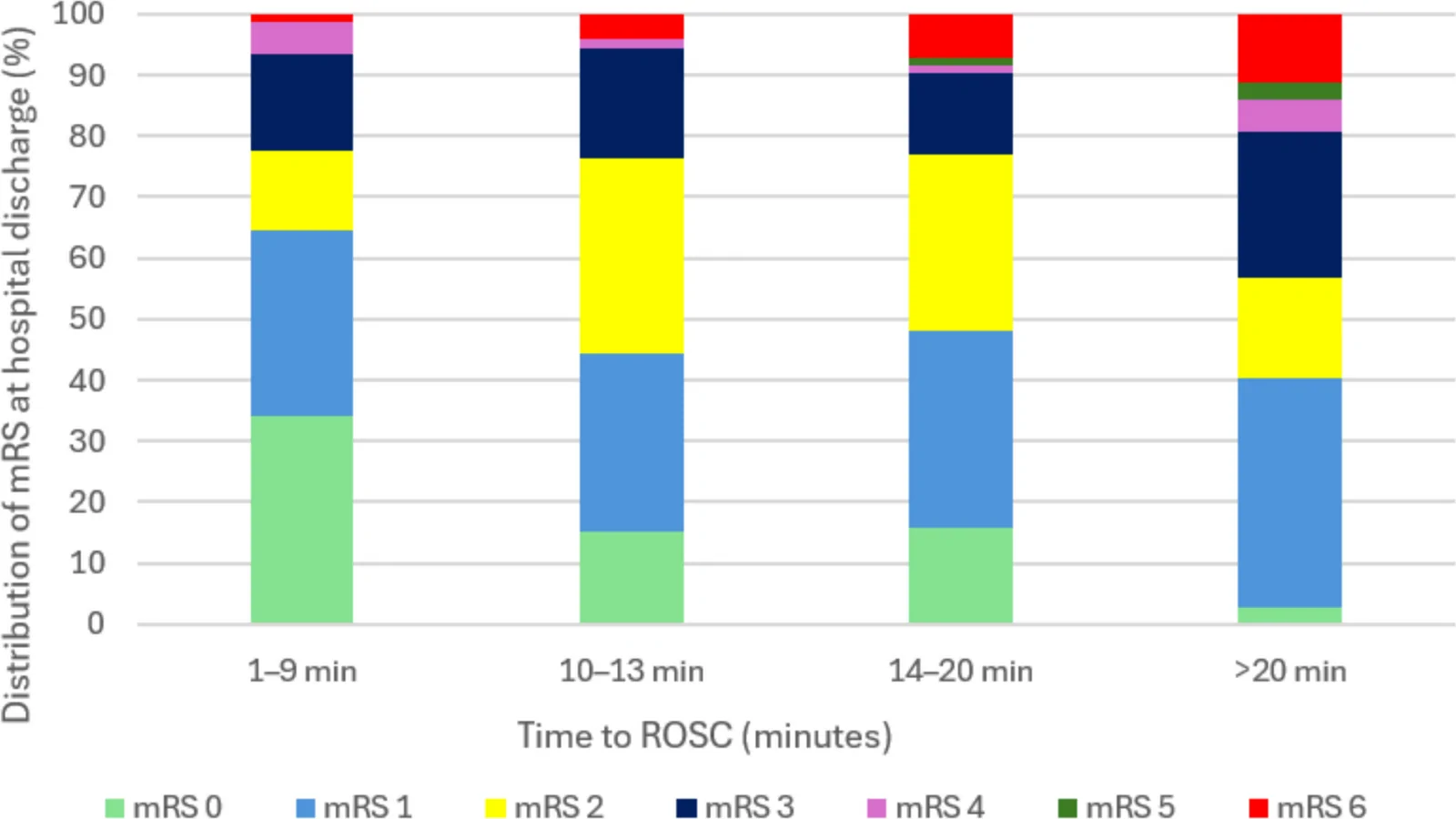
Distribution of mRS scores at hospital discharge stratified by quartiles of time to ROSC (Q1: 1–10 min, Q2: 10–13 min, Q3: 14–20 min, Q4: > 20 min). Abbreviations: mRS, modified Rankin Scale; ROSC, return of spontaneous circulation

To illustrate the association between time to ROSC and neurological outcome at hospital discharge, ROSC times were divided into quartiles and the distribution of mRS scores across these quartiles was visualized (Fig. 1). Across quartiles of time to ROSC, the distribution of mRS scores was significantly different (*p* = 0.002), primarily explained by patients in the highest ROSC quartile (> 20 min), where 43% were assessed within a poor mRS category.

**Fig. 2:**
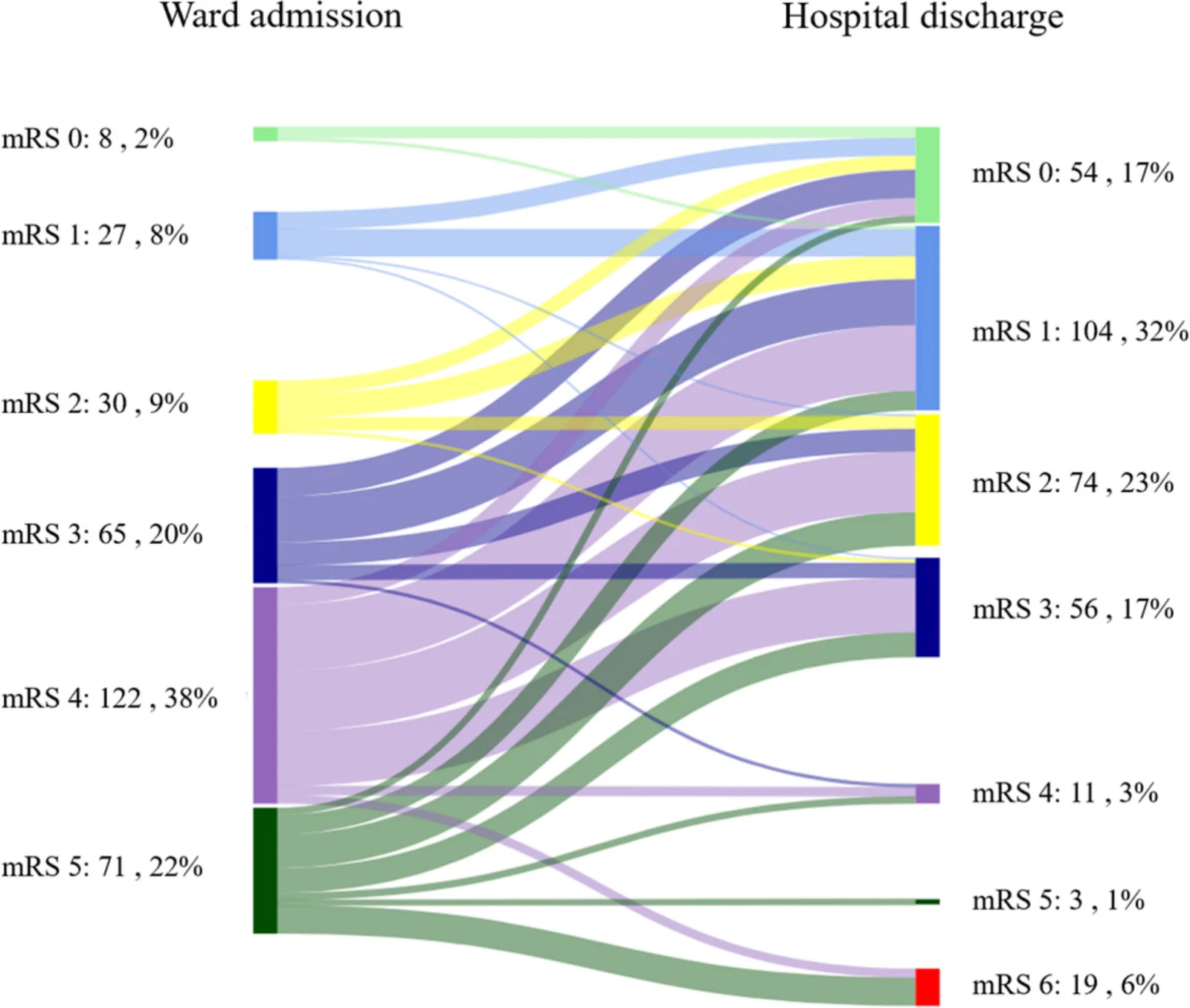
Sankey diagram illustrating temporal changes in mRS scores from admission to the ward and to hospital discharge in patients after OHCA. Abbreviations: mRS, modified Rankin Scale; OHCA, out-of-hospital cardiac arrest

Temporal changes in mRS scores from ward admission to hospital discharge were visualized using a Sankey diagram (Fig. 2). The width of the flows represents the number of patients at each mRS level (0–5 at transfer, 0–6 at discharge). The diagram shows a significant improvement in mRS at hospital discharge in 53% of patients, 20% remained in mRS scores 0–2, whereas 27% remained in mRS scores 3–6, *p* < 0.0001. The diagram illustrates that neurological status is dynamic during the ward stay, with a substantial proportion of patients showing functional improvement between ward admission and hospital discharge.

### Secondary outcome

**Fig. 3:**
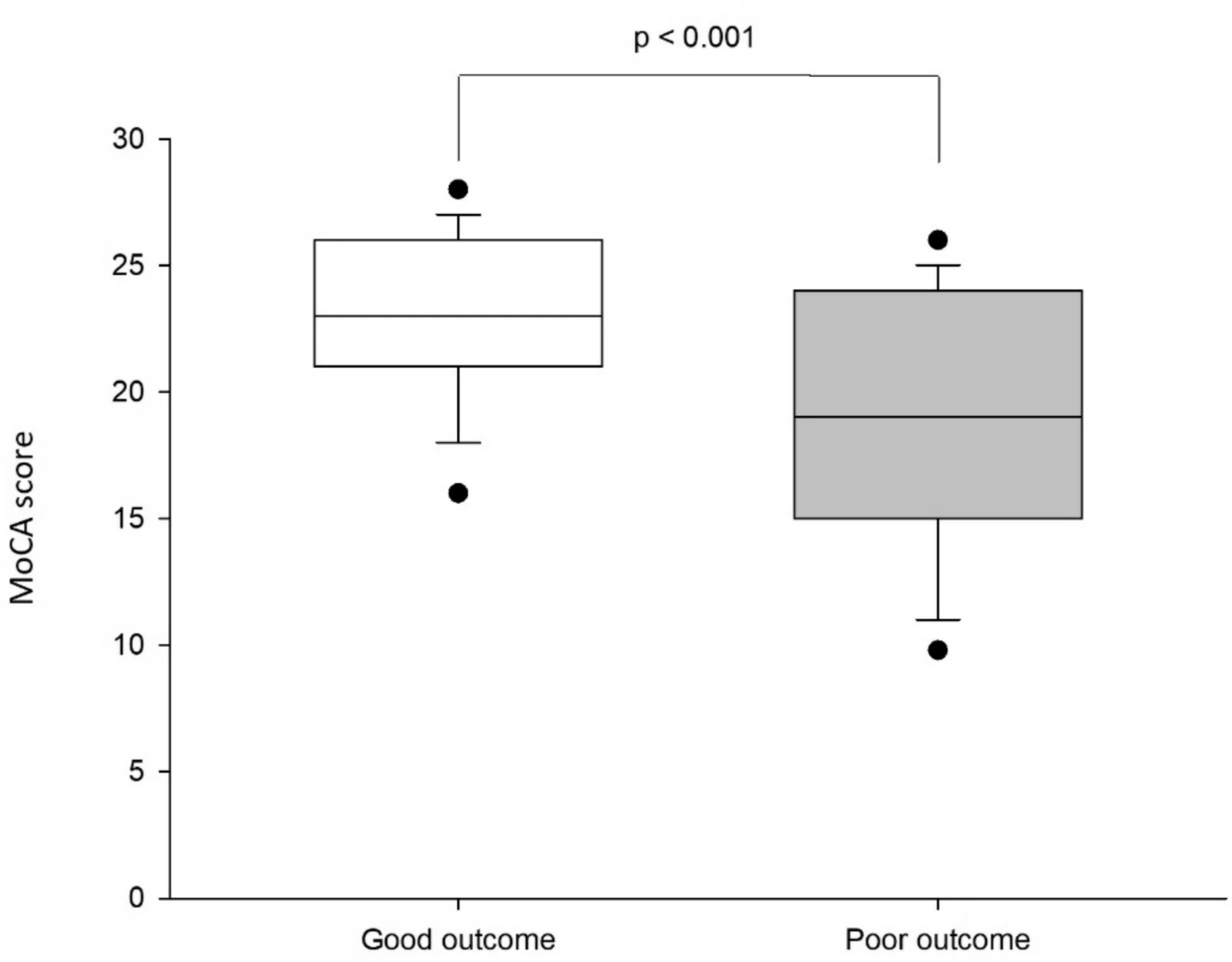
Boxplot of MoCA scores stratified by neurological outcome (Good outcome: mRS 0–2, poor outcome: mRS 3–6). The horizontal line within each box represents median values, boxes extend from the 25th to the 75th percentile of each group’s distribution of values. Whiskers represent 10th to the 90th percentile. • represents the 5th and 95th percentiles. *p*-value represents comparison between groups at each timepoint evaluated by Wilcoxon rank sum test. Abbreviations: MoCA, Montreal Cognitive Assessment; mRS, modified Rankin Scale

MoCA scores were available for 87% of the population with 220 (95%) patients in the good neurological outcome group and 55 (64%) in the poor neurological outcome group. The missing data on MoCA were due to transfer to a different hospital, unable to cooperate, language barrier or deceased before the test could be applied. The good neurological outcome group had a significantly better MoCA score with a median score of 23 (Q25–Q75 21–26) compared to the poor neurological outcome group with a median score of 19 (Q25–Q75 15–24) (*p* < 0.001) (Fig. 3). Among patients with good neurological outcome, 27% were discharged with a MoCA score ≥ 26. MoCA scores were available for 87% of the population, including 95% of patients with good neurological outcome and 64% of those with poor neurological outcome. Missing data were primarily due to inability to cooperate with testing, transfer to another hospital, language barriers, or death prior to assessment.

**Fig. 4:**
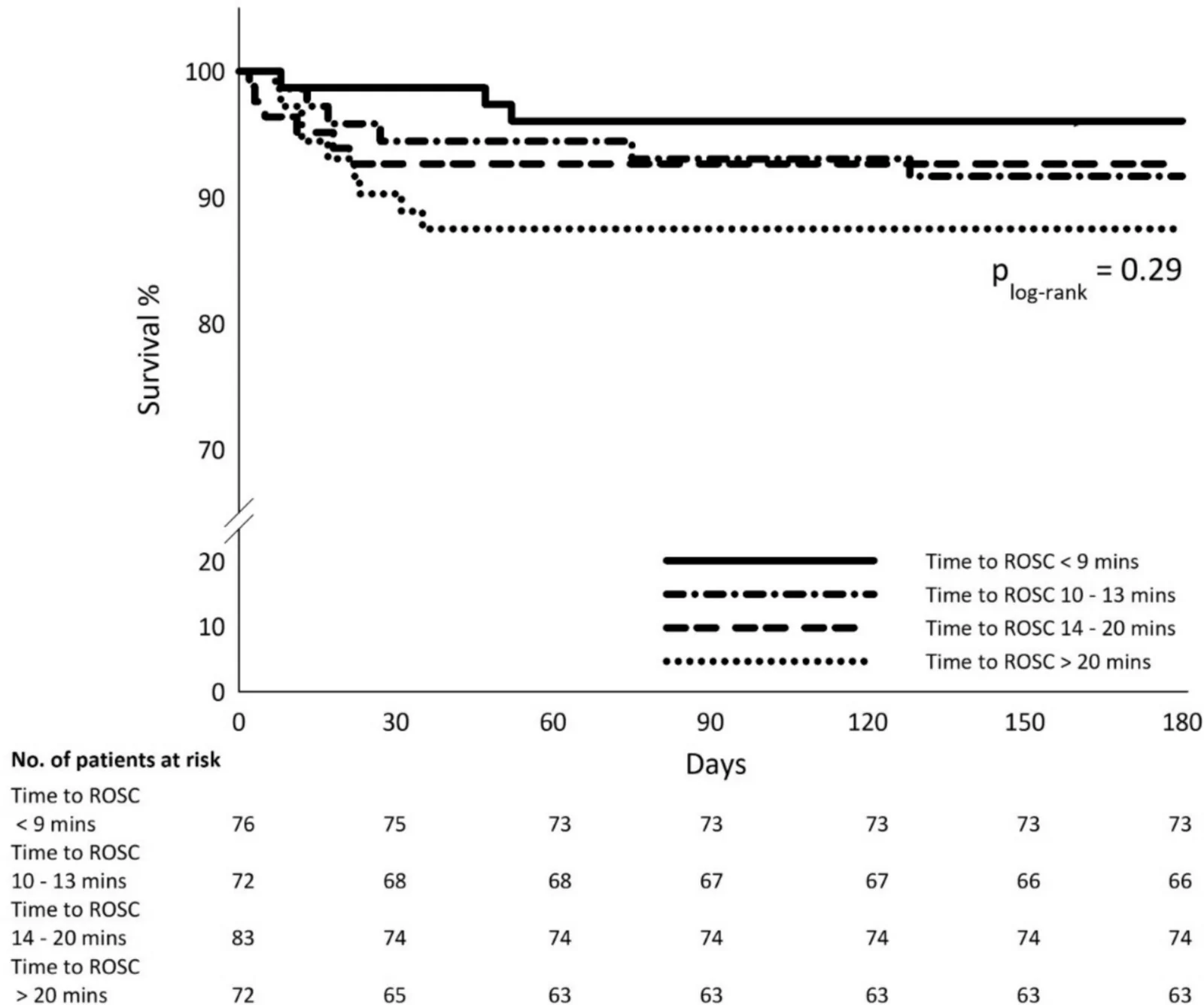
Kaplan–Meier plot displaying 180-day survival stratified according to quartiles of time to ROSC (< 9, 10–13, 14–20, > 20 min). Numbers at risk are shown for each group. Mortality between quartiles was compared with the log-rank test. Abbreviations: ROSC, return of spontaneous circulation

Overall survival at hospital discharge was 94%. Stratifying by quartiles of time to ROSC showed no significant difference in 180-day survival as seen in Fig. 4 (*p* = 0.29). A total of 49 patients (15.3%) died within 180 days, while 272 patients (84.7%) were alive at follow-up. Most deaths occurred early during follow-up, as illustrated by the Kaplan–Meier curves, with few deaths observed later during the follow-up period.

Table 3 provides results of the unadjusted and adjusted Cox proportional hazards analysis. Time to ROSC and low-flow time were not associated with mortality in the unadjusted analysis. Age (HR 1.39, per 5-year increase, 95% CI 1.15–1.68, *p* = 0.0007), shockable rhythm (HR 0.28, 95% CI 0.11–0.70, *p* = 0.007) and no-flow time (HR 1.18 per 1 min, 95% CI 1.07–1.31, *p* = 0.001) were associated with mortality.

**Table 3:** Univariable and multivariable Cox regression analysis of 180-day mortality

	Univariable		Multivariable	
	HR (95% CI)	*p*-value	HR (95% CI)	*p*-value
Age at arrest every 5 years increase	1.39 (1.15–1.68)	0.0007	1.33 (1.07–1.64)	0.009
Gender female vs. male	1.43 (0.58–3.54)	0.44		
Primary rhythm				
Shockable vs. non-shockable	0.28 (0.11–0.70)	0.007		
Other vs. non-shockable	0.71 (0.14–3.49)	0.67		
Witnessed arrest	0.86 (0.26–2.84)	0.80		
Bystander CPR	0.19 (0.08–0.42)	< 0.0001	0.21 (0.09–0.49)	0.002
Normothermia	0.91 (0.43–1.95)	0.81		
Time to ROSC, every 5 min increase	1.08 (0.98–1.19)	0.13		
Low-flow time, every 1 min increase	1.01 (0.99–1.03)	0.30		
No-flow time, every 1 min increase	1.18 (1.07–1.31)	0.001		

Data are presented as hazard ratios (HR) along with their 95% confidence intervals (CI) and *p*-values

In the multivariable analysis, the following variables were entered in the model: age, sex, witnessed arrest, bystander CPR, primary rhythm, and time to ROSC. After applying the backward elimination procedure, the final model consisted of bystander CPR and age at arrest

*CPR* cardiopulmonary resuscitation, *ROSC* return of spontaneous circulation

In multivariable Cox regression analysis, bystander CPR (HR 0.21 (95% CI 0.09–0.49), *p* = 0.002) and age (HR 1.33, per 5-year increase, (95% CI 1.07–1.64), *p* = 0.009) were independently associated with mortality.

## Discussion

In this study addressing early neurological function, we found that increasing time to ROSC was independently associated with a 12% decrease in the odds of a good neurological outcome for every 5-min increase, however no association with mortality was found. Neurological function improved significantly during admission with the majority (72%) having a good neurological outcome at hospital discharge. Patients with good neurological outcome also demonstrated significantly better cognitive functions with 27% having a MoCA score within the normal range.

The association between time to ROSC and neurological outcome remained significant after adjusting for age and primary rhythm. Furthermore, no significant interaction was identified as part of the model control. Age and primary rhythm are well-established predictors of neurological outcome after OHCA [4] and also showed strong associations in our study. These factors may partly explain the relationship between time to ROSC and neurological outcome, as patients with a shockable rhythm often achieve ROSC earlier and generally have a more favorable prognosis. However, the persistence of the association after adjustment suggests that time to ROSC captures additional clinically relevant information related to the duration and effectiveness of resuscitation efforts. The credibility of this association is increased by the results on the univariate analysis of the no-flow and low-flow times, where we found decreased odds of a good neurological outcome by 12% and 2%, respectively. These numbers show the critical importance of timely resuscitation efforts in improving not only acute survival but also early neurological outcomes in post-arrest care and support that rapid initiation of CPR by bystander is a key factor in preventing and determining early neurological recovery among OHCA survivors [4].

A similar study by Iqbal et al. [12] included 174 OHCA patients who achieved ROSC and was admitted to a specialized cardiac center. They reported duration of resuscitation attempt to be independently associated with neurological outcome, with an OR of 0.95 per minute increase. The discrepancy in odds ratio compared to the current study (14% decrease per 5 min), may be explained by a slight difference in definition of good neurological outcome (mRS 0–3 vs. mRS 0–2).

The study by Matsuyama et al. [13] included 23,803 patients and showed a decreasing likelihood of good neurological outcome with increasing CPR duration, with the first 20 min being particularly important. They used Cerebral Performance Category which is a method for assessing neurological function and has been shown to correlate with mRS [14]. Despite differences in inclusion criteria and methodology, both studies demonstrate a clear association between time to ROSC and neurological outcome.

A retrospective cohort study by Reynolds et al. [15] included 1014 patients with 11% surviving to hospital discharge. 6% achieved a favorable neurological outcome (mRS 0–3, same as in Iqbal et al. [12]). They found that the probability of favorable outcome declined significantly with increasing time to ROSC, with almost 90% of favorable cases achieving ROSC within 16 min. These findings are consistent with our results and further support the likelihood of favorable neurological recovery declining significantly beyond 15–20 min of resuscitation.

A significant difference in MoCA performance was observed between patients with good and poor neurological outcomes, with lower scores in the poor outcome group. Even within the good neurological outcome group, the majority of patients demonstrated MoCA scores below the cut-off considered normal, indicating that cognitive impairment remains common despite good functional recovery. These findings align with those of Wagner et al. [16] reporting similar MoCA levels among hospitalized OHCA survivors, supporting the use of both mRS and MoCA as complementary instruments for the evaluation of neurological outcomes after cardiac arrest. However, a recent study by Brouer et al. [17] found no association between time to ROSC and MoCA, suggesting that our observed link between time to ROSC and neurological outcome mainly reflects non-cognitive aspects captured by the mRS.

Time to ROSC was not significantly associated with 180-day survival in initial survivors. This indicates that while time to ROSC is still a key determinant of survival and neurological function, its significance for in-hospital and 180-day mortality may be limited in this selected population. Our study confirmed that other known factors such as age, bystander CPR, no-flow time and type of primary rhythm may still be important in terms of survival. In a study by Kjaergaard et al. [3], they found a 2% increase in mortality for every minute increase in time to ROSC. This study included only unconscious patients admitted to intensive care, whereas our study included only survivors at admission to the cardiology ward.

Lastly, across quartiles of time to ROSC, most deaths occurred early during follow-up, as illustrated by the Kaplan–Meier curves, with few deaths observed later in the follow-up period. This highlights the importance of the initial recovery phase addressing the etiology of cardiac arrest and initiation of secondary prophylactic initiatives.

### Strengths and limitations

As for strengths of the study, the patients were prospectively included in a well-defined regional OHCA registry, and data were analyzed retrospectively. This design ensures systematic data collection while minimizing selection and recall bias. To address potential biases in subjective assessments and ensure the validity of our data, we calculated a Cohen’s kappa score of 0.73, indicating substantial inter-observer agreement in assessing neurological outcome. Furthermore, multivariable analyses were performed to adjust for potential confounding factors, thereby supporting the validity of the observed associations.

There may be important limitations to this study. Our study population consisted exclusively of patients who survived the initial resuscitation phase and were transferred to the cardiology ward. This conditional inclusion may introduce survivorship bias, as patients with prolonged time to ROSC who died early were not part of the current population. Consequently, the absence of association between time to ROSC and 180-day mortality likely reflects the lack of early high-risk individuals prior to cohort entry. The mortality findings should therefore be interpreted within this selected survivor population and not extrapolated to a general OHCA population. The study was conducted at a single tertiary center and included only surviving patients admitted to the cardiology ward after OHCA with presumed cardiac cause, which limits the generalizability of the findings to other healthcare settings, patient populations, and non-cardiac OHCA. The retrospective cohort study design presents some inherent challenges such as the information on exposure and neurological outcomes being associative which means we cannot establish causality. Neurological outcomes were assessed using mRS scores derived primarily from review of medical records rather than direct patient interviews.

MoCA testing could not be performed in all patients and was less frequently available in those with poor neurological outcome, most commonly due to inability to cooperate with testing or death prior to assessment. This likely introduces informative absence and may underestimate the degree of cognitive impairment in the overall cohort. Therefore, the MoCA findings should be interpreted as exploratory. In addition, MoCA was assessed once during hospitalization, close to discharge, and scores may therefore be influenced by factors such as fatigue, mood, or the acute recovery phase following cardiac arrest. Lastly, premorbid neurological status was not systematically assessed in this study. Some patients may therefore have had pre-existing neurological impairment, such as prior stroke or dementia, which could influence the likelihood of achieving a favorable neurological outcome. However, in Denmark, patients with severe frailty or advanced neurological disease are often subject to advance decisions regarding resuscitation, which may reduce the number of such patients receiving resuscitation attempts and thereby included in the study population.

## Conclusion

In initial OHCA survivors transferred to the cardiology ward, increased time to ROSC was independently associated with decreased odds of good neurological function at hospital discharge, even after adjusting for age and primary rhythm. The majority of patients (72%) had good neurological function at hospital discharge. In this population time to ROSC was not associated with mortality.

## Data Availability

The data that support the findings of this study are not publicly available due to restrictions related to Danish data protection legislation and patient confidentiality but are available from the corresponding author upon reasonable request.
